# The mARS complex: a critical mediator of immune regulation and homeostasis

**DOI:** 10.3389/fimmu.2024.1423510

**Published:** 2024-06-21

**Authors:** Sharon Bright Amanya, Damilola Oyewole-Said, Keenan J. Ernste, Nalini Bisht, Arnav Murthy, Jonathan Vazquez-Perez, Vanaja Konduri, William K. Decker

**Affiliations:** ^1^ Department of Pathology and Immunology, Baylor College of Medicine, Houston, TX, United States; ^2^ Department of Natural Sciences, Rice University, Houston, TX, United States; ^3^ Dan L. Duncan Comprehensive Cancer Center, Baylor College of Medicine, Houston, TX, United States; ^4^ Center for Cell and Gene Therapy, Baylor College of Medicine, Houston, TX, United States

**Keywords:** aminoacyl-tRNA synthetase (aaRS) complexes, immune regulation, homeostasis, immune disease, eukaryote biology

## Abstract

Over the course of evolution, many proteins have undergone adaptive structural changes to meet the increasing homeostatic regulatory demands of multicellularity. Aminoacyl tRNA synthetases (aaRS), enzymes that catalyze the attachment of each amino acid to its cognate tRNA, are such proteins that have acquired new domains and motifs that enable non-canonical functions. Through these new domains and motifs, aaRS can assemble into large, multi-subunit complexes that enhance the efficiency of many biological functions. Moreover, because the complexity of multi-aminoacyl tRNA synthetase (mARS) complexes increases with the corresponding complexity of higher eukaryotes, a contribution to regulation of homeostatic functions in multicellular organisms is hypothesized. While mARS complexes in lower eukaryotes may enhance efficiency of aminoacylation, little evidence exists to support a similar role in chordates or other higher eukaryotes. Rather, mARS complexes are reported to regulate multiple and variegated cellular processes that include angiogenesis, apoptosis, inflammation, anaphylaxis, and metabolism. Because all such processes are critical components of immune homeostasis, it is important to understand the role of mARS complexes in immune regulation. Here we provide a conceptual analysis of the current understanding of mARS complex dynamics and emerging mARS complex roles in immune regulation, the increased understanding of which should reveal therapeutic targets in immunity and immune-mediated disease.

## Introduction

Over the course of evolution, organisms have become increasingly complex. The transition from simple unicellular organisms to complex multi-cellular organisms created key functional challenges specifically in the ability to maintain homeostasis in response to change. To adapt to increasing demands, individual proteins within the cells of multicellular organisms have undergone extensive structural changes to take on unique roles (1, 2). For example, aminoacyl tRNA synthetases (aaRS), enzymes that catalyze the attachment of amino acids to their cognate tRNA during translation, have acquired multiple, novel domains over the course of evolutionary history (3, 4).

In bacteria, the aaRS exhibit a simple core structure comprised of class-specific catalytic and anti-codon binding (ACB) domains that mediate the tRNA aminoacylation function (5). Some bacterial aaRS also contain an editing domain for deacylating mischarged tRNAs (5). In addition to these base structural domains, eukaryotic aaRS have added new domains and motifs at their N or C-termini or inserted into their protein cores (4). These new domains play a myriad of ex-translational roles that regulate cellular homeostasis. Through these new domains and motifs, aaRS may assemble into large multi-subunit complexes to enhance the efficiency of biological functions. Moreover, the complexity of the multi-aminoacyl tRNA synthetases (mARS) complexes increase with complexity of the organisms from which they are isolated, suggesting contributions to the maintenance of cellular homeostasis in multicellular organisms. While limited evidence suggests the presence of aaRS complexes in bacteria, Harrris reported a large multi-aminoacyl tRNA synthetase complex comprised of IleRS, TyrRS, GluRS, and SerRS in *Escherichia coli*. Interestingly, the size of the complex was reported to vary depending on the method of cell lysis used, with a 400 kDa complex seen following sonication but a 1MDa complex seen using the freeze press method (6). Unfortunately, no other study has been able to validate or build upon these findings.

In eukaryotes, the existence of mARS complexes has been extensively reported with increasing complexities described from yeast to mammals. In the archaea *Methanothermobacter thermautotrophicus*, the mARS complex is comprised of ArgRS and SerRS that associate via binding of ArgRS to the SerRs C-terminal domain (7, 8). The complex is reported to enhance efficiency of aminoacylation, particularly under conditions of extreme salt concentration and temperature, thereby suggesting a major homeostatic role in thermo-osmoadaptation (7). In the yeast *Saccharomyces cerevisiae*, MetRS forms a complex with GluRS and Arc1p via N-terminal domains to form a GST-like fold (9, 10). In addition to enhancing the efficiency of aminoacylation, this complex regulates the switch from fermentation to respiratory metabolism in yeast. During respiratory adaptation, the Snf1/4 glucose-sensing pathway inhibits Arc1p expression, triggering the simultaneous release of GluRS and MetRS. Free MetRS translocates to the nucleus where it regulates transcription of ATP synthetase genes. GluRS on the other hand translocates to the mitochondria where it mediates the translation of mitochondrial ATP synthase genes (11, 12). Therefore, Arc1P acts as a cytosolic anchor of MetRS and GluRS from which they are released following the specific cellular cues that signal a need for differential regulation of cellular metabolic processes. In the parasite *Tryopanasoma brucei*, there exists a 1.2 MDa mARS complex comprised of six aaRS (MetRS, ProRS, GlnRS, AlaRS, TrpRS, and AspRS) and three MARS complex-associated proteins (MCP1, MCP2, and MCP3) (13). Interestingly, Cestari reported that the composition of the complex varied depending on the life cycle stage of the parasite. The mARS complex isolated from the blood form is comprised of MetRS, ProRS, TrpRS and MCP1, while in the procyclic form, MCP1, GlnRS and MCP2 were additionally identified. While the authors attributed the difference in composition to experimental limitations rather than valid biological differences, these compositional changes could indeed also arise from differences in cellular regulatory processes required by metabolic and synthetic requirements of the different parasite forms. Just like in yeast, mARS complexes in *T. brucei* enhance tRNA-aminoacylation via binding of tRNA to MCP2. Indeed, conditional repression of MCP2 led to reduced parasite growth and infectivity, highlighting the key role played by MCP2 and the mARS complex in regulating parasite fitness (13).

**Figure 1:**
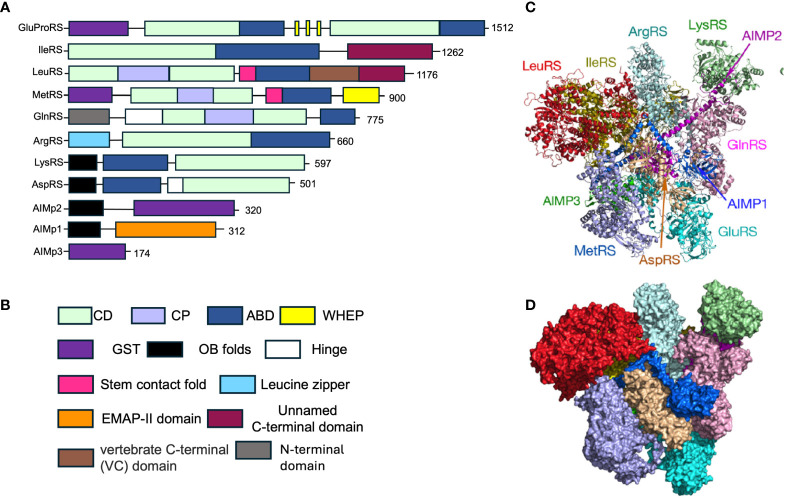
Structure and composition of the mammalian mARS complex. **(A)** Domains that constitute the individual aaRS and aaRs interacting multi-functional proteins (AIMPs) of the mARS complex. Each color represents a specific domain as follows: Green-catalytic domain, lavender-connective polypeptide, navy blue-antibody binding domain, yellow-WHEP domain, purple-GST-like domain, black-Oligonucleotide binding fold, white-Hinge, pink-stem contact fold, blue-leucine zipper, orange-EMAP-II domain, brown-vertebrate c-terminal domain, Gray-N-terminal domain, and maroon-unnamed c-terminal domain. **(B)** Legend for the domains. **(C)** Ribbon and **(D)** hollow structures of the mARS complex as reported by Khan et al, 2020 (republished with permission).

In mammalian cells, the mARS complex is a 1.2 MDa protein complex canonically comprised of eight aaRS including MetRS, AspRS, LysRS, ArgRS, LeuRS GlnRS, IleRS, the fused GluProRS and three aaRS-interacting multi-functional proteins known as AIMp1, AIMp2, and AIMp3 (**Figure 1**) (14, 15). Several appended domains including GST-like domains, zinc finger domains, leucine zippers, and oligonucleotide binding (OB) folds (**Table 1**, structures and biological function obtained from InterPro database, ref. 16) within the aaRS contribute to the protein-protein interactions that enable mARS complex formation (14, 17). Moreover, these newly acquired domains and motifs are not critical for tRNA charging but instead contribute to non-canonical functions of the aaRS, extensively reviewed in Guo (2010) and in Smirnova (2012) (4, 18). While mARS complexes in lower eukaryotes enhance the efficiency of aminoacylation, sufficient evidence to support a similar role in mammalian cells is lacking. Rather, subunits of the mARS complex are reported to regulate various cellular processes including angiogenesis, apoptosis, inflammation, metabolism, and immune regulation, among others (19–21).

**Table 1:** New appended domains of aaRS and aaRS interacting multi-functional proteins.

Domain	Structure	mARS subunit	Other proteins	Biological function
WHEP	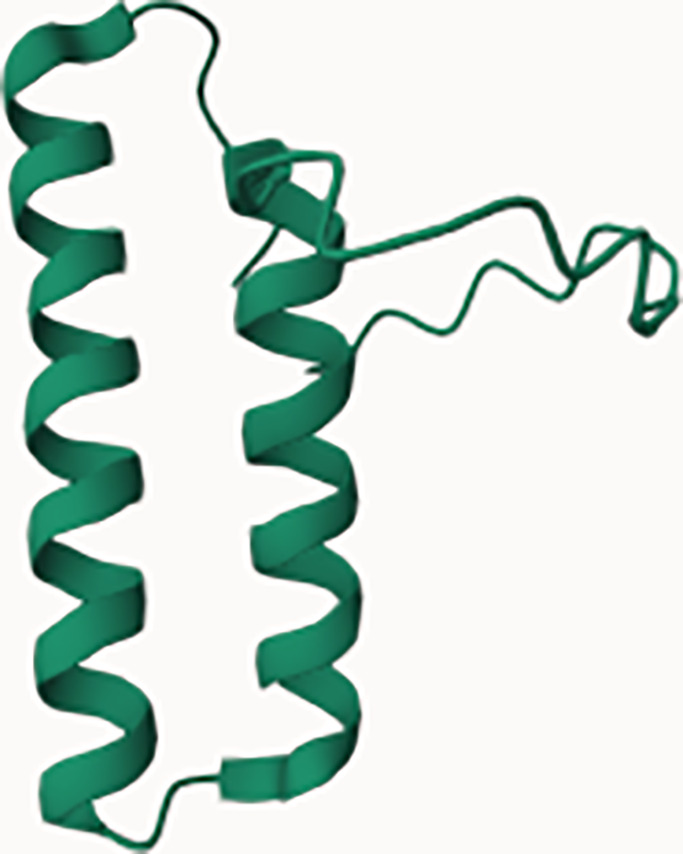	GluProRS MetRS	HisRS TrpRS GlyRS	Association of aaRS into the mARS complex
GST	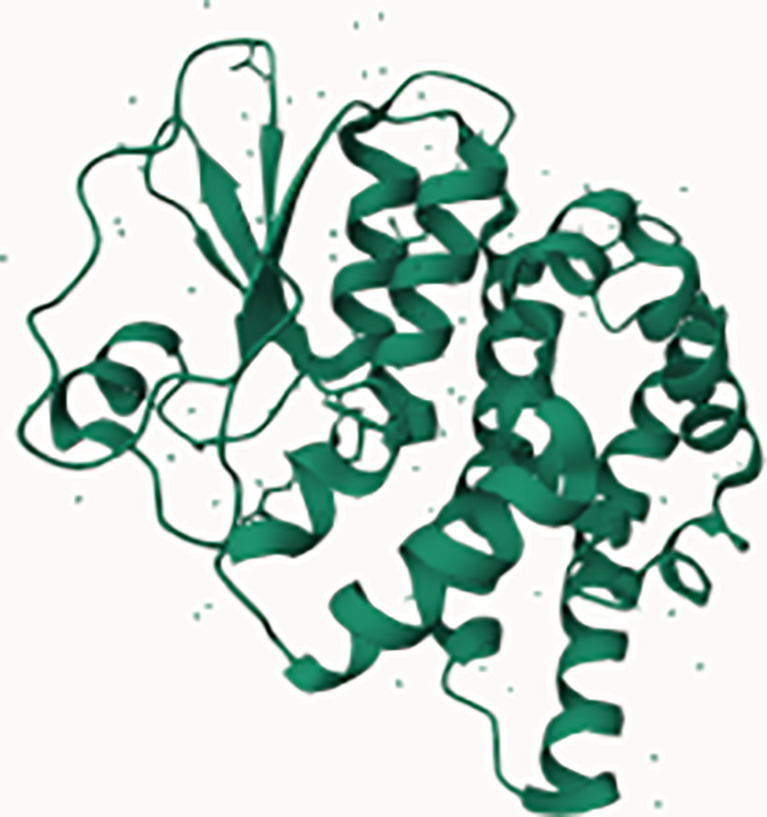	MetRS GluProRS AIMP2 AIMP3	Eukaryotic elongation factor-1 gamma (eFF1G) Hsp26	Detoxification of reactive electrophilic compounds by catalyzing their conjugation to glutathione
Leucine Zipper	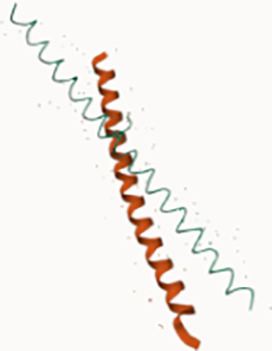	ArgRS AIMP1 AIMP2	Transcription factors; FOS & JUN NFAT GCN4 ATF4-C/EBP BETA	Proteins that contain LZ mediate sequence specific DNA-binding followed by a leucine zipper region for dimerization e.g. transcription factors binding to promoter regions
Oligonucleotide/oligosaccharide binding motif (OB fold)	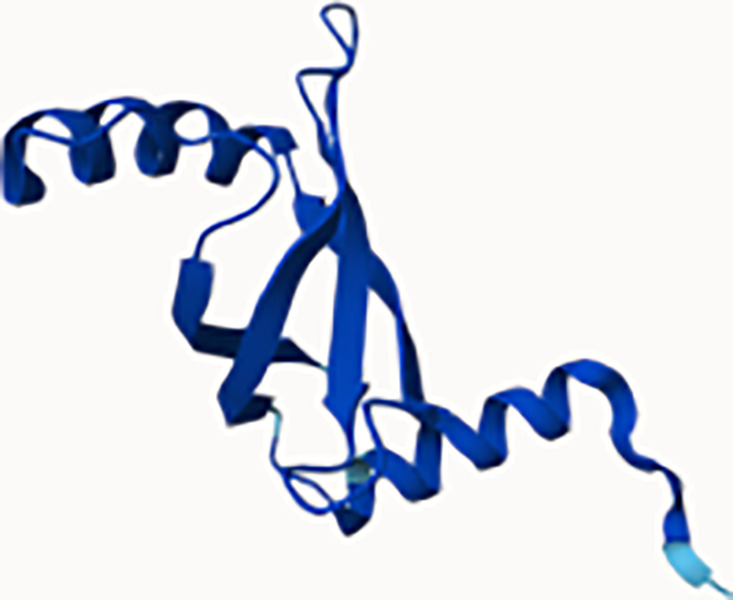	LysRS NTD AspRS NTD AIMp1 AIM2 2	Nucleic acid binding proteins e.g. ssDNA-binding proteins (CDC13) Phase ssDNA-binding proteins (gp32, gp25, gpV) Cold shock proteins DNA ligases RNA capping enzymes DNA replication initiators RNA polymerase subunit RBPB	Bind nucleic acids Present in exonuclease IV as 2 identical subunits. ExoVII is a single strand-specific exonuclease which degrades ssDNA from both 3’and 5’ ends Plays a role in methyl-directed mismatch repair and may guard the genome from mutagenesis by removing excess ssDNA since the buildup of ssDNA could induce SOS and PolIV dependent mutagenesis
Zinc Finger Domain	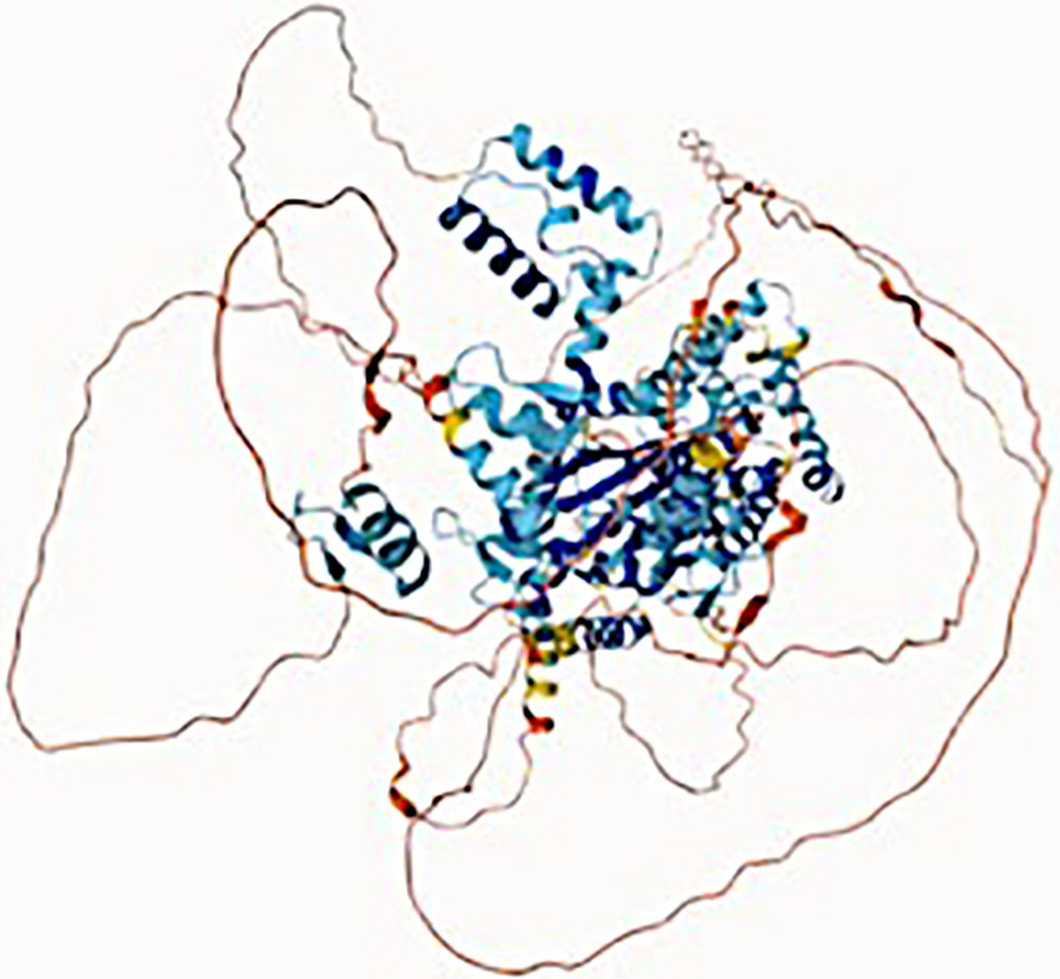	IleRS (c-terminus)	C-terminus of DNA glycosalase/AP Lysase enzymes	Base excision repair of DNA damaged by oxidation or by mutagenic agents

Release of aaRS isoforms from mARS complexes is often mediated by post-translational modifications (PTM). For example, GluProRS dissociates from the mARS complex following interferon gamma (IFN-γ)-mediated phosphorylation at Ser 886 and Ser 999 in its linker region (22). Following release, GluProRS interacts with other proteins to form the gamma activated inhibitor of translation (GAIT) complex which mediates IFN-γ-induced suppression of proinflammatory genes. Similarly, LysRS is released from the mARS complex upon phosphorylation at Ser 207 (23), and once released, translocates to the nucleus where it binds the transcription regulator microphthalmia-associated transcription factor (MITF) and regulates target gene expression. Further, nuclear ArgRS may dissociate from the mARS complex to associate with SRRM2, a component of the nuclear splicing machinery (24) impacting expression and alternative splicing of protein-coding transcripts. Because mARS complex subunits regulate a myriad of cellular processes which appear critical to immune homeostasis, it would be instructive to examine the role of such complexes in immune cell settings. To this end, this review presents not only a thorough exegeses of the current evidence identifying immune cell immunomodulatory roles of the mARS complex but it identifies key investigative questions within this paradigm that could cement the mARS complex as an indispensible regulator of immune function.

## Methods

A comprehensive narrative review was conducted using PubMed with a focus on the role of the mARS complex in immune regulation and pathogenesis of immune-related diseases. The search terms included “mARS complex”, “MSC”, “aaRS”, “AIMP”, “evolution”, “immune disease”, and “autoimmunity” with inclusion of all research papers published in the English language by December 2023. The review provides a critical analysis of: 1) the immunoregulatory roles of immune cell mARS complexes, 2) immunoregulatory roles of mARS complexes in immune-related cells, and 3) the role of the mARS complex in the pathogenesis of immune-related diseases. We further raises new questions, the answers to which will advance our understanding of the pivotal role of the mARS complex in immune regulation and potentially as a druggable target in immune-related diseases.

### Immunoregulatory roles of immune cell mARS complexes

**Figure 2:**
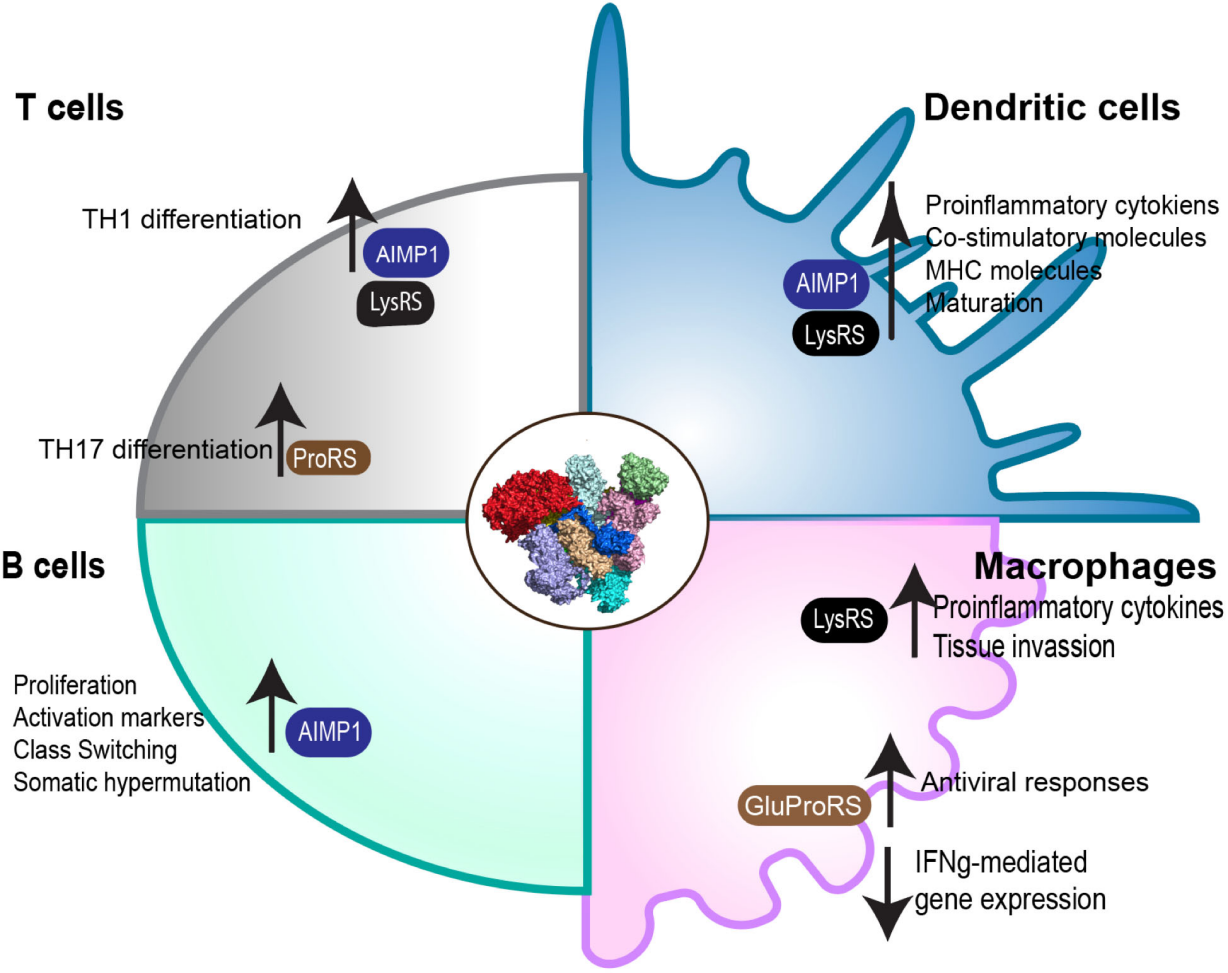
Role of the mARS complex in immune cell function. Through its subunits, the mARS complex plays distinct roles in the regulation of differentiation and function in a variety of different immune cells.

mARS complexes regulate various processes in immune cells which are critical for their differentiation and function (**Figure 2**).

#### Dendritic cells

Dendritic cells (DC) are innate, antigen-presenting cells with the ability to prime and activate naïve T-cells (25–27). In this capacity, DC provide a link between the innate and adaptive arms of the vertebrate immune response. Immature DC reside in peripheral tissues where they surveil the environment for immunologic danger signals, specifically pathogen-associated molecular patterns (PAMPs) and damage-associated molecular patterns (DAMPs). Upon encountering such signals, DC undergo maturation - a process characterized by the upregulation of major histocompatibility complex (MHC) antigen presentation molecules, co-stimulatory molecules that include CD40, CD80, CD83, CD86, and others, cytokine secretion (28, 29), and chemokine receptor expression (30). Mature DC then migrate to the peripheral lymphoid organs and present their most-recently sampled antigens to T-cells (30).

In DC, the mammalian mARS complex has been reported to take on unique features and roles pertinent to DC maturation and polarization. We have previously demonstrated that the composition of amino acyl tRNA synthetases within the complex may change depending upon the amino acid sequence composition of antigenic peptides presented by DC (31). If MHC class I and class II antigenic peptides share a stretch of identical amino acid homology, amino acyl tRNA synthetases of the corresponding, cognate amino acids join the complex, including those that are not canonically associated with mARS complexes. This peptide homology-mediated change in mARS complex composition is accompanied by the polarization of DC towards a T helper type 1 (T_H_1) phenotype characterized by increased production of IL-12 and dissociation of AIMp1 from the mARS complex (31). Multiple studies have highlighted the T_H_1-polarizing effects of AIMp1 on DC. We have elaborated on the critical role of AIMp1 in T_H_1 immunity through characterization of AIMp1^-/-^ mice (32–34). Bone marrow-derived DC (BMDC) differentiated from AIMp1^-/-^ mice show significantly reduced proinflammatory cytokine expression including IL-12, IL-6, and IL-1β compared to WT BMDC following treatment with lipopolysaccharides (LPS). Moreover, T-cells co-cultured with AIMP^-/-^ DCs exhibited low levels of IFN-γ production. The loss of AIMp1 also impaired expression of CD86 and CD40 co-stimulatory molecules and p38 MAPK signaling in mature DC, resulting in a reduced ability of these cells to prime T-cells for anti-tumor and anti-viral responses (32). Moreso, analysis of data from GEO and TGCA databases demonstrated that elevated AIMp1 expression positively correlated with an increase in activated tumor-infiltrating DC and a T_H_1 T-cell signature, and expression levels of AIMp1 were much better correlated with increased survival in melanoma, ovarian cancer, and breast cancer than were levels of IL-12 or IFN-γ expression, the mild correlations of which were insignificant (32).

DC maturation is a critical step that precedes its ability to activate T-cells. Following recognition of danger signals by pattern recognition receptors (PRRs), signaling cascades drive transcriptional and proteomic changes within DC. This reorganized transcriptome and proteome constitute DC polarization, the phenotype of which subsequently influences the identity and character of the downstream T_H_ response. Of the many signaling pathways involved, p38MAPK, mTORC1, NFkB and AP-1 are the most widely studied (35–40) and several subunits of the mARS complex regulate these pathways. For example, LysRs induces maturation and activation of DC through the MAPK and NFκB pathway (41). Kim reported that treatment of DC with lysRS leads to phosphorylation of a series of MAPK effectors including JNK, p38, and ERK as well as degradation of IκB, an inhibitory protein that prevents NFκB translocation into the nucleus. Consequently, LysRS treatment induces NFκB nuclear translocation where NFκB regulates genes important for DC polarization (41). Moreover, sequential inhibition of p38MAPK and NFκB demonstrated that inhibitors of MAPK effectors restored Iκα and Iκβ, suggesting the involvement of MAPK as an upstream regulator of NFκB in the LysRS-mediated maturation and activation of DC (41). In another study, AIMp1 treatment enhanced NFκB binding to the TLR2 promoter and modulated gene expression in DC (42), highlighting the role of NFκB in regulating transcriptional changes induced by mARS complex subunits. There exists additional evidence suggesting regulation of p38/MAPK by AIMp1. Inhibition of p38/MAPK suppressed the ability of DCs to produce IL-12 and consequently the ability to induce differentiation of IFN-γ-producing T-cells. Additionally, AIMp1 dissociation from the mARS complex influences the function of PP2A, a phosphatase that negatively regulates p38/MAPK activity. This observation was further validated in AIMp1^-/-^ mice in which both p38/MAPK and the BMDC T_H_1 polarizing gene signature were substantially inhibited (32).

Upon maturation, DC adopt a transcriptional program characterized by increased expression of MHC antigen presentation complexes, co-stimulatory molecules (CD40, CD80, and CD86) and pro-inflammatory cytokines including IL-12, TNF, and IL-1β. Several studies have shown that various subunits of the mARS complex including AIMp1 and LysRS can upregulate this transcriptional signature in DC (32, 41, 43). Similarly, other aaRS including ThreRS (44), TrpRS (45), and TyrRS (46), not canonically known to associate with the mARS complex, have also been shown to regulate this transcriptional signature. Lastly, LysRS through its regulation of AP_4_A synthesis has been shown to increase motility and antigen presentation functions of dendritic cells. As expected, BMDC from *Nudt2^fl/fl^*/CD11c-cre mice in which AP_4_A synthesis is enhanced due to the absence of Ap4A hydrolase prime a significantly stronger CD8^+^ T-cell responses highlighting the role of LysRS in regulating not only motility and antigen presentation, but functional activation of T cells as well (47). The roles of other subunits of the mARS complex in regard to DC function however remain elusive and therefore future studies will be critical to advance our understanding of this area.

#### Macrophages

Macrophages are tissue-resident antigen-presenting cells that play critical roles in maintaining tissue homeostasis. They mediate elimination of pathogen and damaged cells while coordinating tissue repair and remodeling. Depending on tissue microenvironment, macrophages can be polarized toward M1 or M2 subtypes, each subset having a unique role (48). M1 macrophages, also known as classically activated macrophages are characterized by the production of TNF, IL-1, IL-6, IL-12, and IL-23 following stimulation by IFNγ, GM-CSF, and LPS (48, 49). The polarization of macrophages toward the M1 phenotype is regulated by several signaling pathways including NFκB, AP-1, interferon-regulatory factor (IRF)-5, and signal transducer and activator of transcription 1 (STAT1) (50–53). These macrophages are critical for anti-viral, anti-bacterial, and anti-tumor immune responses due to their ability to produce microbiocidal and tumoricidal substances including nitric oxide (NO) and reactive oxygen species (ROS). Conversely, M2 macrophages, also known as alternatively activated macrophages, upregulate the anti-inflammatory cytokine IL-10 and downregulate the proinflammatory cytokine IL-12. M2 macrophages also exhibit increased production of arginase-1 (Arg-1), an enzyme known to deplete L-arginine thereby impairing T-cell activation and function. Macrophages become polarized toward the M2 phenotype through a controlled signaling cascade involving STAT6, IRF-4, peroxisome proliferator-activated receptor (PPAR)-γ, and cAMP-responsive element-binding protein (CREB) (24, 54). M2 macrophages are implicated in the propagation of chronic inflammation, tumorigenesis, and metastasis (48, 55), and therefore maintenance of tissue homeostasis generally requires tight regulation of M1/M2 polarization.

Several subunits of the mARS complex have been implicated in the regulation of macrophage polarization. For instance, LysRS expressed by colon cancer cells induces the polarization of tumor-associated macrophages (TAMs) toward the M2 phenotype which in turn activates cancer cells and cancer-associated fibroblasts (CAFs) to regulate metastasis (56). LysRS-positive cancer cells secrete cytokines including GAS6, IL-8, and ANG which can reprogram M1 macrophages toward an M2 phenotype, thereby facilitating subsequent tumor infiltration (56). This study however did not report on the role of endogenous LysRS in macrophage polarization. Another study reported that LysRS induces the production of proinflammatory cytokines from macrophage-like THP-1 cells following activation by Shiga toxin (Stx) (57). Shiga toxins are virulence factors produced by *Shigella dysenteriae* and human pathogenic Shiga toxin-producing *Escherichia coli* (STEC). The toxin is comprised of two subunits, subunit A which has enzymatic activity, and subunit B which is critical for receptor-mediated entry. The enzymatic Stx subunit A has been shown to induce LysRS dissociation from the mARS complex and its subsequent secretion from differentiated THP-1 macrophage-like cells. Indeed, co-treatment of THP-1 cells with Stx plus purified LysRS enhanced the production of proinflammatory cytokines much more than each alone; suggesting that Stx may mediate its proinflammatory effects by inducing release of LysRS which may then act in an autocrine fashion to propagate inflammation (57). Interestingly, GluProRS has been shown to inhibit translation of proinflammatory cytokines in macrophages following IFNγ treatment (58). IFN-γ induces a series of signaling events that lead to the activation of cyclin-dependent kinase (Cdk-5) and its regulatory protein Cdk5R1(p35) which phosphorylate GluProsRS at Ser886 and induce downstream phosphorylation at Ser999. This signaling event leads to the release of GluProRS from the mARS complex, and its association with the GAIT complex. During GAIT assembly, NSAP1 interacts with GluProRS to form the pre-GAIT complex after which other partners join to form the active GAIT complex. GluProRS in the GAIT complex then directly binds the 3’UTR GAIT element on target mRNAs while P-L13a interacts with elF4G of the translation initiation complex to block assembly of small ribosomal subunits and consequently the initiation of translation (22, 58). Through these mechanisms, the mARS complex manipulates translation of specific genes that regulate macrophage phenotype.

Several signaling pathways are involved in the regulation of macrophage phenotype, and mARS complex subunits have been reported to play key roles in this process. LysRS activates p38MAPK to induce TNF expression and cell migration, and LysRS treatment of macrophages upregulates production of matrix metalloproteinase 9 (MMP-9) to aid in tissue invasion (59). Additionally, Glu-ProRS regulates antiviral immunity in macrophage-like U937 and RAW264.7 cells (60). Upon viral infection, GluProRS undergoes infection-specific phosphorylation at Ser990 which induces its dissociation from the mARS complex and participation in functions distinct from its known roles in the GAIT complex. GluProRS is a positive regulator of the RIG-I and MDA5-mediated type I interferon pathway and acts downstream of mitochondrial antiviral signaling protein (MAVS) and upstream of TRAF3. GluProRS interacts with PCB2, a negative regulator of MAVS which is known to trigger he ubiquitination and degradation of MAVS after viral infection. MAVS is a key protein in a signaling cascade important for anti-viral immune responses. The GluProRS-PCBP2 interaction blocks PCBP2-mediated ubiquitination of MAVS which then propagates the antiviral signaling cascade that suppresses viral replication (60). Moreover, GluProRS knockdown reduced antiviral responses demonstrated by lowering IFN-γ and IL-6 production following viral infection or treatment with synthetic double-stranded RNA poly(I:C) (60). As expected, stable overexpression of GluProRS in these cells rescued the antiviral phenotype and upregulated innate antiviral immunity against RNA viruses. GluProRS heterozygotes exhibited higher levels of influenza viremia, produced lower levels of the inflammatory cytokines IFN-β and IL-6, and delayed viral clearance in comparison to GluProRS wildtype animals (60). The ability of GluProRS to play distinct roles depending on cellular cues demonstrates the role of the mARS complex and indeed its subunits in sensing and maintaining homeostasis. The mechanism(s) through which cellular cues are transmitted to the mARS remain to be investigated.

LysRS also regulates antiviral pathways, particularly in response to RNA: DNA hybrids. LysRS binds and sequesters RNA: DNA hybrids that arise as a consequence of chronic inflammation, thereby slowing recognition and subsequent activation of cGAS-STING (61). During chronic inflammation, DNA from nearly any source (tumor cells, dead cells, viruses, microorganisms) are sensed by cGAS, leading to its activation. Activated cGAS then synthesizes cyclic guanosine monophosphate-adenosine monophosphate (cGAMP) as a secondary messenger which then binds to STING (stimulation of interferon genes). Subsequently, cGAMP-bound STING migrates from the endoplasmic reticulum to the golgi apparatus where it recruits and activates protein kinases TBK1 and IKK, the upstream initiators of the IRF3 and NFκB inflammatory pathways. LysRS inhibits these proinflammatory processes in two important ways, namely 1) through its N-terminal domain that interacts with RNA: DNA hybrids to delay recognition by cGAS, therefore impeding cGAMP production and 2) through LysRS-dependent production of diadenosine tetraphosphate (AP4A), a negative regulator of STING-dependent signaling. Through these complementary mechanisms, LysRS leads to resolution of chronic inflammation induced by nucleic acid ligands (61).

#### T-cells

T-cells are a specialized group of lymphocytes that mature in the thymus. They are broadly classified into two groups, CD4^+^ T cells and CD8^+^ T-cells, both of which play key roles in adaptive immune responses. CD4^+^ T cells canonically play helper and regulatory roles although cytotoxic functions have also been reported (62, 63). CD4^+^ helper T cells are divided into several subsets namely Th1, Th2, Th17, and T_REG_ cells based on the cytokines they produce as well as their functional characteristics (64). T_H_1 cells secrete IFN-γ and TNF to increase Type-1 immune responses including macrophage activation, B-cell activation, and cytotoxic T-cell responses. These have extensively been reviewed elsewhere (65–67). T_H_1 cell polarization occurs following naïve CD4^+^ T-cell recognition of antigen presented by APCs secreting the cytokine IL-12. IL-12 receptor signaling in T-cells activates transcription factors including T-bet and STAT4, regulating T_H_1-associated genes that mediate differentiation into the T_H_1 cell phenotype (68–70). T_H_2 cell polarization is mediated by APCs secreting IL-4 which mediates the upregulation of the GATA3 and STAT6 transcription factors (71). These regulate the expression of the T_H_2 gene signature, which is characterized by the production of IL-4, IL-13, and IL-5. T_H_2 responses are important in mediating allergy and defense against eukaryotic pathogens. T_H_17 differentiation is driven by APC secretion of IL-6 and TGF-β, driving the expression of the RORγt transcription factor and production of IL-17 and IL-22 (72, 73). T regulatory cells (Tregs) act to suppress immune responses to maintain homeostasis and self-tolerance. Tregs can be categorized as “natural”, developing in the thymus during negative selection, or “induced” by tolerogenic antigen presentation in the periphery. Regulatory T cells express the Foxp3 transcription factor and are characterized by the production of the tolerogenic cytokine IL-10 (74). CD8^+^ T cells on the other hand, are generally associated with T_H_1 immunity and known for their cytotoxic function (75, 76). CD8^+^ T-cells are activated by APC presentation of antigens via MHC class I in conjunction with IL-12 secretion. Activated CD8^+^ T-cells kill target cells through directed release of cytotoxic molecules including granzyme B, perforin, and IFN-γ and are critical for antitumor and antiviral immunity (77–80).

Through its subunits, the mARS complex regulates several aspects of T-cell biology including activation, differentiation, and effector functions. The mARS component AIMp1 enhances T_H_1 immunity when released and secreted. Kim et al. demonstrated that mice treated with a DNA adjuvant encoding a fusion protein that linked an anti-CD3 single chain Fv to AIMp1 significantly upregulated T_H_1 immune responses as characterized by increased IFN-γ production in CD4^+^ T-cells and increased levels of IgG_2a_ with concomitant functional inhibition of T_H_2 immunity (81). Interestingly, a contrasting study suggested that in CD4^+^ T-cells, AIMp1 may drive T regulatory cell differentiation rather than T_H_1 immune responses (82). Here, AIMp1 inhibited T-cell receptor (TCR)-dependent activation by reducing lipid raft association in AIMp1 treated cells. Accordingly, the reduced lipid raft association limited the formation of TCR-centered molecular activation clusters and downstream activation (82). TCR stimuli-induced calcium influx was then reduced in addition to phosphorylation of downstream signaling molecules PLCγ and PI3K when CD4^+^ T-cells were cultured in presence of AIMp1 (82). This result may have been model dependent and its physiologic relevance remains unclear. Additional work should resolve these conflicting reports.

ProRS, another subunit of the mARS complex which is present as GluProRS, is reported to mediate T_H_17 differentiation. Febrifugine, a bioactive compound in many Chinese medicinal herbs mediates its tolerogenic effects by inhibiting the pro-T_H_17 function of ProRS. Halofuginone (HF), a derivative of febrifugine, competes with proline for binding to the ProRS tRNA binding site, leading to the accumulation of uncharged tRNA^pro^. The accumulation of tRNA^pro^ activates the amino acid response pathway (AAR) characterized by GCN2 autophosphorylation and induction of the AAR-response gene DIT3. DIT3 regulates cellular responses to stress including inhibition of T_H_17 differentiation (83, 84). The inhibitory effects of halofuginone on ProRS can be reversed by supplementation of proline or GluProRS (83, 84). Indeed, treatment with HF has been shown to reverse T_H_17-mediated multiple sclerosis in mouse models (83). Further, GluProRS also may join the GAIT complex following its release from the mARS and regulate the expression of proinflammatory genes (58). Lastly, the expression of LysRS in tumor-associated immune cells (TAIs) including macrophages/monocytes and CD4^+^ T cells correlated with longer overall survival in patients with gastric carcinomas (85). Interestingly, the expression of LysRS in tumor cells was correlated with poor prognostic parameters including larger tumor size, higher Ki-67 proliferation index, increased vascular invasion, and overall shorter survival (85). How LysRS in TAIs mediates antitumor effects remains a subject of investigation.

#### B-cells

B cells are mediators of adaptive humoral immunity through their role as antigen-presenting cells and through the production of antibodies directed against self or non-self antigens. Mature B-cells recirculate within secondary lymphoid organs in search of their cognate antigens. Once these antigens are encountered, B cells process, internalize, and present them to helper T-cells which are critical for germinal center formation, class switching, affinity maturation, and the development of memory (86). More recently, regulatory B-cells have been described as a small subset of B cells that have immune inhibitory characteristics similar to those of T regulatory cells (87, 88). While limited data exist on the role of the mARS complex or its subunits in B-cells, Kim et al. (2015) reported AIMp1 as a novel B-cell activator via stimulation of the PKC-NFκB pathway (89). AIMp1 increases B-cell activation and proliferation as demonstrated by increased expression of activation markers including CD86, CD69, and MHC class II. AIMp1 also increased the expression of activation-induced deaminase (AID), an enzyme critical for somatic hypermutation and class switching as demonstrated by elevated levels of antigen-specific IgG_1_, IgG_2a/_IgG_2b_, IgG_3_, and IgE (89).

### Immunoregulatory roles of the mARS complex in immune-related cells

#### Microglia

Microglia are tissue resident macrophages of the central nervous system and provide primary immune surveillance of the brain. Microglia respond to DAMPs or PAMPs by generating proinflammatory cytokines and presenting phagocytosed antigens. Depending on the stimuli, microglia can differentiate into pro-inflammatory M1 state or anti-inflammatory M2 state. M1 microglia secrete proinflammatory cytokines including IL-6 and TNFα and are linked to neuroinflammation and severe CNS disease. In contrast, M2 microglia are anti-inflammatory and maintain basal brain immune homeostasis (90, 91). Just as in other immune cell types, subunits of the mARS complex modulate microglial function. Such subunits include AIMp1 which promotes activation and proinflammatory functions of microglia. Kim et al. (2022) demonstrated that treatment of microglia with AIMp1 led to M1 polarization with in*c*reased production of proinflammatory cytokines including IL-6, IL-1β, and TNF. Additionally, CD86, MHC class II, and the M1 specific marker CD68 were also upregulated (92). JNK and p38 MAPK pathways were the upstream regulators of AIMp1-induced microglial cell activation, and pharmacologic inhibition of JNK and/or p38MAPK significantly reversed the M1-polarizing effects of AIMp1 (92).

Endothelial monocyte-activating polypeptide II (EMAP II), a proinflammatory cytokine generated by cleavage of the AIMp1 C-terminal domain, is highly expressed in microglia during neurodegenerative conditions including neurotoxic lesions (93) and status epilepticus (94). Similarly, ArgRS has been reported to play a role in microglial activation during ischemic stroke (95). Moderate microglial activation following stroke induces the scavenger role of clearing cellular debris whereas overactivated microglia induce production of proinflammatory molecules including cytokines, nitric oxide, and reactive oxygen species (ROS) (96, 97). This leads to recruitment of other immune cells which orchestrate nonspecific innate immune responses, significantly exacerbating ischemic injury. In a rat model of ischemic brain injury, ArgRS knockout alleviated the hyperactivated phenotype of microglia and provided neuroprotective effects by sparing mitochondrial morphological and functional integrity from ischemic insult, thus attenuating brain injury (95).

#### Osteoclasts

Osteoclasts are bone-resorbing cells that differentiate from the monocyte/macrophage lineage upon exposure to macrophage colony stimulating factor (M-CSF) and receptor activation of NFκB ligand RANKL (98, 99). Because of their bone-degrading functions, osteoclasts are involved in various bone pathologies including osteoporosis, bone tumors, and Paget’s disease (100). Certain subunits of the mARS complex have been shown to regulate aspects of osteoclast differentiation and function. For example, AIMp1 has been reported to be a novel oncogene in multiple myeloma, promoting osteoclastogenesis and contributing to osteoclastogenesis-induced multiple myeloma (101). Multiple myeloma is a hematological malignancy characterized by abnormal clonal plasma cells in the born marrow with potential for uncontrolled growth which may cause destructive bone lesions, kidney injury, and hypercalcemia. The interaction of myeloma cells with the bone microenvironment activates osteoclasts while suppressing osteoblasts, thereby leading to bone loss (102, 103). Wei et al. (2022) reported that AIMp1 mediates pathology in multiple myeloma by interacting with ANP32A, a histone acetyltransferase, to promote the histone acetylation enrichment function of GRB2-associated and regulator of MAPK protein 2 (GAREM2) and increase activation of the MAPK signaling pathway (101). ANP32A expression was increased in patients with MM, and increased expression was correlated with reduced survival. Further, treatment with AIMp1 activated the nuclear factor of activated T cells c1 (NFATc1) to mediate osteoclast differentiation, suggesting that several signaling pathways are involved in AIMp1-driven multiple myeloma. Indeed, AIMp1 levels were elevated at both the transcript and protein level in MM patients and were associated with decreased survival and elevated Ki-67 expression (101). Similarly, Hong et al. (2015) reported that AIMp1 induced osteoclastogenesis *in vitro* and was elevated in rheumatoid arthritis (RA) patients (104). As such, AIMp1 has been evaluated as a therapeutic target both in MM and RA. Wei et al. (2022) demonstrated that siAIMp1-loaded exosomes can suppresses MM and reduce osteoclast differentiation both *in vitro* and *in vivo (* 101) while another group demonstrated that targeting AIMp1 with a monoclonal antibody atliximab inhibited AIMp1-mediated osteoclastogenesis *in vitro* and significantly reduced disease severity in a mouse model of collagen-induced arthritis (104). While AIMp1 presents a promising target in the treatment of several immune-related diseases, we still have open questions on how this might affect the mARS complex dynamics and cellular homeostasis. More studies are needed to address these key questions to ensure the success of AIMp1 targeting therapies in clinical development. Additionally, there is growing evidence that IleRS plays a role in osteoclast-mediated osteoporosis. The small molecule inhibitor reveromycin A (RM-A) has been shown to mediate anti-osteoporosis effects by blocking aminoacylation activity of IleRS. While signaling pathways directly affected by IleRS aminoacylation activity remain to be determined, the authors demonstrated that RM-A treatment induced osteoclast cell death and associated bone resorption (105, 106).

#### Mast cells

Mast cells are tissue resident cells which play a critical role in responding to eukaryotic pathogens through the release of proinflammatory mediators following surface crosslinking of Fcε3R1 receptors (107). An extensive literature exists on the role of the mARS complex in the regulation of mast cell function through its LysRS subunit. Following FcεR1 aggregation, the MAPK pathway is activated leading to the phosphorylation of LysRS at ser207. In the mARS complex, LysRS is a dimer that binds to the N-terminal domain of AIMp2 via its dimer interface. Because ser207 is located at the dimer interface, its phosphorylation provokes structural changes that disrupt binding of LysRS to AIMp2 and induce release from the larger complex (108). Once released, LysRS drives synthesis of diadenosine tetraphosphate (AP_4_A) and binds to the transcription factor Microphthalmia-associated transcription factor c (MITF) in the MITF-Hint complex. Through this mechanism, FcεR1 aggregation leads to elevated expression of MITF-responsive genes such as mast cell protease (mMCP-6 & mMCP-5), p75 nerve growth factor, granzyme B, and tryptophan hydroxylase, all critical for mast cell development and function (23, 109). Hint is a negative regulator of MITF, and qPCR assay of MITF-controlled genes including RMCP-6, c-kit receptor tyrosine kinase, lymphocyte serine protease granzyme B, and tryptophan hydroxylase demonstrated all were elevated with mediated accumulation of AP4A after receptor crosslinking. Therefore, through the synthesis of AP4A in stimulated mast cells, LysRS regulates transcriptional activity (23). Additionally, LysRS through its induction of AP4A synthesis also regulates other transcription factors within the MITF family including USF2. USF2 is a ubiquitously expressed transcription factor in eukaryotic cells and plays critical roles in cell growth and survival. Lee et al. (2005) demonstrated that USF2, Hint, and LysRS form a multiprotein complex in mast cells and that increased AP4A induced by LysRS dissociates Hint from this complex (110). Because Hint is a negative regulator of USF2, dissociation of Hint relieves the inhibitory effect, facilitating expression of USF2-responsive genes including telomerase catalytic subunit (TERT), protein tyrosine phosphate 1 (SHP), and transforming growth factor β2 (TGF-β2). Indeed, the introduction of exogenous Ap4A enhanced the expression of these USF2-regulated genes in mast cells (110). This interaction has been exploited by HIV-1 for regulation of its own genes. USF2 is one of the transcription factors that bind the HIV-1 viral promoter in the 5’ long terminal repeat (5’ LTR) to drive expression of genes regulating HIV-1 replication and latency. Tang et al. (2023) demonstrated that ser207 phosphorylation-mediated release of LysRS from the mARS complex facilitates HIV infection by upregulating USF2 activity including transcription of viral genes that facilitate HIV replication (111). Overexpression of the AIMp2 N-terminus, a peptide that stabilizes the LysRS association with the mARS complex, inhibited HIV-1 replication along with formation of proviral DNA and other USF2-controlled genes (111).

### The mARS complex and immune disease

Several subunits of the mARS complex play important roles in the mediation of various autoimmune conditions. For example, AIMp1 has been implicated in the pathogenesis of systemic lupus erythematosus (SLE) and glomerulonephritis as well as multiple myeloma through its regulation of gp96 localization (112). Gp96 is an endoplasmic reticulum (ER)-resident chaperone protein which belongs to the HSP90 family. It is continuously recycled to the ER by COPI-coated vesicles with ER localization mediated by its C-terminal KDEL sequence recognized by KDEL receptors in the ER. Surface expression of gp96 mediates the activation and maturation of dendritic cells by binding to its receptor CD91 (113–115), and AIMp1 regulates both ER localization and cell surface localization of gp96. Han et al. (2007) demonstrated that AIMp1 can be co-purified with gp96 together with the COPI complex in the microsomal compartment. This interaction is facilitated by AIMp1 amino acids 54–192 and C-terminal amino acids 699–799 of gp96 near its KDEL motif (34). The KDEL endoplasmic reticulum protein retention receptor 1 (KDEL1) is a receptor important in the retention of soluble ER-resident proteins including gp96. The authors determined that AIMp1 also co-purified with KDELR-1 in addition to gp96, suggesting a possible role in gp96 ER trafficking. Indeed, gp96 was found to localize in the perinuclear ER in WT cells while it localized to the plasma membrane in AIMp1^-/-^ cells (34). Previous reports show that gp96 increased the activation and maturation of dendritic cells via binding to CD91 (114, 115). Because AIMp1 regulates ER-targeting of gp96, thereby reducing its plasma membrane localization, it was unsurprising that AIMp1 knockout upregulated gp96 plasma membrane localization together with DC maturation and activation. Further, AIMp1 knockout increased gp96 plasma membrane expression and was associated with autoimmune disorders such as systemic lupus erythematosus and lupus nephritis (34). Lupus nephritis is a severe complication of SLE that arise when immune complexes formed by autoantibodies accumulate or are deposited in the glomeruli. These immune complexes trigger inflammation via complement and Fc-depended pathways which result into nephritis characterized by hematuria and proteinuria (116). Elevated serum levels of AIMp1 have been reported in SLE patients and are predictive of active disease (117). Treatment with AIMp1 targeting antibody atializumab significantly reduced the severity of nephritis symptoms including proteinuria, glomerular damage, and renal deposition of immune complex in lupus-prone mice (NZB/NZW) (118). Atializumab also reduced proinflammatory cytokines including IFN-γ, IL-17A, and IL-6. In contrast, anti-inflammatory responses that included upregulation of IL-10 secreting Tregs were significantly increased by atializumab treatment. As AIMp1 is reported to mediate proinflammatory responses via NFκB, its inhibition by atializumab predictably suppressed NFκB activation by inhibiting Ikβα degradation (118).

In another study, the authors demonstrated that hepatitis C virus (HCV) induced liver fibrosis and autoimmune disease via its membrane protein E2 interaction with AIMp1 (112). AIMp1 was co-purified with HCV E2, and addition of E2 reduced AIMp1 protein expression in a dose dependent manner, suggesting a direct regulatory role of E2 on AIMp1. AIMp1 transcription measured by qPCR was not affected by E2, however ubiquitination of AIMp1 was increased by addition of E2 suggesting that HCV E2 targets AIMp1 for proteasomal degradation. Because AIMp1 is known to reduce gp96 surface expression by ER targeting, the authors found that E2-mediated reduction of AIMp1 led to increased surface expression of gp96. Moreover, TGF-β signaling as well as TGF-β-controlled genes, known to be negatively regulated by AIMp1, were also elevated in E2 treated cells which suggested a multi-faceted mechanism underlying HCV mediated liver fibrosis and autoimmunity (112).

GluProRS has been shown to orchestrate multiple myeloma pathogenesis. Karuta et al. (2023) demonstrated that GluProRs is critical for growth and survival of multiple myeloma (MM) cells and is an excellent therapeutic target in MM treatment. In this study, levels of aminoacyl tRNA synthetases between normal plasma cells and MM cells were evaluated. While aaRs overall were elevated in MM cells in comparison to normal plasma cells, only GluProRS was both strongly upregulated and associated with poor clinical outcomes. In MM cells, the EPRS gene that encodes for GluProRS is often amplified and is a risk factor for development of MM. Moreover, small molecule inhibitors (NCP26, HFG, & ProSA) targeting ProRS exert anti-proliferative effects on all MM cell lines tested. Studies that demonstrate the precise mechanism by which ProRS inhibitors mediate anti-proliferative effects in MM will be helpful toward improving the understanding of how aaRS and other mARS complex subunits contribute to the orchestration of immune disease.

AIMp1 has also been implicated in the pathogenesis of osteoarthritis, with AIMP1 significantly elevated in chondrocytes isolated from osteoarthritis patients (119). While highly flexible, cartilage rarely recovers from osteoarthritis-induced injury given its limited supply of blood, nutrients, and nervous tissue (120). Accordingly, stimulation of chondrocyte proliferation and extracellular matrix synthesis has become an attractive treatment strategy since chondrocytes synthesize joint-supporting cartilage, type II collagen, and proteoglycans. This observation highlights the need to understand factors that regulate dedifferentiation and degeneration of chondrocytes, a critical hallmark of osteoarthritis. One of these factors is TGF-β, a widely reported regulator of chondrogenic differentiation and degeneration (121–123). The TGF-β signaling pathway regulates a variety of cellular processes including plasticity, proliferation, differentiation, apoptosis, and migration. Initiation of the cascade through binding of TGF-β to cell surface receptors TGFBRI and TGFBR2 leads to formation of receptor heterodimer, the active form of which phosphorylates the R-Smad proteins Smad2 and Smad3. Smad2/3 form a heterodimer complex with co-Smad4, and the complex translocates to the nucleus to govern expression of TGF-β-regulated genes. TGF-β signaling can be inhibited by targeted proteasomal degradation of Smurf2, a TGFβ-targetingE3 ubiquitin ligase (124). Kim et al. (2008) reported that AIMp1 inhibits TGF-β signaling by stabilizing Smurf2, thereby increasing TGF-β receptor degradation. Indeed, depletion and knockdown of AIMp1 enhanced phosphorylation of Smad2/3 and increased the expression of TGF-β target genes p27, p15, and PAI-1. To understand the mechanism underlying the regulatory functions of TGF-β signaling, the interaction between AIMp1 and Smurf2 was further investigated. AIMp1 co-purified with Smurf2, with specific binding of AIMp1 amino acids 193–312 to the Smurf2 WW domains. AIMp1 binding stabilized Smurf2 and consequently enhanced TGF-β receptor ubiquitination and proteasomal destruction (125). Since AIMp1 negatively regulates TGF-β, inhibition of AIMp1 has been studied as a potential therapeutic target in osteoarthritis and cartilage injury. Ahn et al. (2016) reported that AIMp1 negatively regulated TGF-β signaling via interaction with Smad2/3 and that increased expression of AIMp1 significantly increased osteoarthritis in patent-derived degenerated chondrocytes compared to healthy controls (119). Moreover, the localization of AIMp1 was also shown to change with chondrocyte pathology. Whereas AIMp1 localized to the nucleus in healthy chondrocytes, in dedifferentiated chondrocytes, AIMp1 localization shifted to the cytoplasm. Downregulation of AIMp1 by siRNA targeting significantly increased TGF-β signaling and led to redifferentiation of both dedifferentiated and degenerated chondrocytes. These findings were validated in mouse models in which the authors demonstrated that knockdown of AIMp1 resulted in enhanced cartilage tissue formation in both dedifferentiated and degenerated chondrocytes through induction of type II collagen (119).

## Conclusion

mARS complex regulation of various immune cell signaling events, phenotype, and gene regulation implies a deeper role of the mARS in immune homeostasis than previously understood or appreciated. From roles in regulating myeloid cell and helper T-cell polarization to regulation of B-cell functions like class switching and somatic hypermutation, the mARS complex through its subunits asserts itself as critical to the governance of immune homeostasis. Moreover, mARS also regulates activation, differentiation and function of various tissue specialized immune cells including osteoclasts, microglia and mast cells. It is therefore unsurprising that mARS has been implicated in the pathogenesis of immune-related diseases including systemic lupus erythematosus, nephritis, multiple myeloma, tumorigenesis, and osteoarthritis. For example, the subunit AIMp1 regulates the localization of gp96, a chaperone belonging to the HSP90 family that is critical for dendritic cell activation and Th1 immune responses. When AIMp1 is dysregulated, gp96 is targeted to the cell membrane surface where it triggers Th1 immune responses including the auto-antibodies involved in the pathogenesis of systemic lupus erythromatosus and lupus nephritis. GluProRS on the other hand facilitates proliferation and survival of multiple myeloma cells and therefore critical for its pathogenesis. Further, AIMp1-mediated inhibition of TGF-β signaling in chondrocytes propagates osteoarthritis pathogenesis. This central role in immune governance positions the mARS complex and its subunits as candidates for therapeutic development to combat immune-related diseases. However, accomplishing this will require additional investigation into the following outstanding questions.

1) mARS complex subunits have acquired new domains over the course of evolution. These include GST-like domains, zinc finger domains, leucine zippers, and oligonucleotide binding (OB) folds which are homologous to domains in non-related proteins. These perform a myriad of functions including transcription, DNA repair, mRNA splicing, and protein-protein interactions. Whether subunits of mARS complex that poses these domains have capacity for similar roles remains an open question.
2) AIMp1 has been shown to be a critical signaling component of the mARS complex that dissociates following distinct cellular cues. What post translational modification(s) trigger its release and how does this impact broader mARS complex dynamics?
3) Subunits of the mARS complex including ArgRS, AIMp1, and MetRS regulate processes in various cellular compartments including the cytoplasm, mitochondria, and nucleus. What cues trigger localization in these compartments and the localization signals that facilitate targeting?
4) Some subunit of the mARS complex such as AIMp1, LySRS and LeuRS are secreted, performing their regulatory roles in a paracrine fashion. What is the secretory pathway(s) through which these subunits are transported and to which receptors do they bind?
Answering these outstanding questions will not only advance our knowledge of mARS complex-mediated immune regulation but also aid in identifying druggable targets key to the development of next-generation therapies for immune-related diseases.

## Author contributions

SA: Conceptualization, Project administration, Writing – original draft, Writing – review & editing. DO-S: Conceptualization, Writing – review & editing. KE: Conceptualization, Writing – review & editing. NB: Conceptualization, Writing – review & editing. AM: Conceptualization, Writing – review & editing. JV-P: Conceptualization, Writing – review & editing. VK: Conceptualization, Writing – review & editing. WD: Conceptualization, Funding acquisition, Project administration, Supervision, Writing – review & editing.
